# Needle Aspiration Versus Catheter Drainage in Medium-Sized (5-10 cm) Liver Abscesses: A Comparative Study in Mumbai, India

**DOI:** 10.7759/cureus.38240

**Published:** 2023-04-28

**Authors:** Solai Muthu Rajagopal S, Girish Bakhshi, Gayatri A Muley

**Affiliations:** 1 General Surgery, Grant Government Medical College and Research Institute, Mumbai, IND

**Keywords:** entamoeba histolytica, needle aspiration, catheter drainage, percutaneous drainage, image guided therapy, liver abscess

## Abstract

Background

Liver abscess is a disease known to mankind since ancient times and has been treated by various means. The introduction of radiology-guided interventional drainage procedures has reduced the mortality rate, which forms a significant part of management. However, there is still a dilemma regarding the procedure of choice in medium-sized liver abscesses mainly in resource-limited developing countries.

Methods

The study was conducted on 60 patients with moderate-sized (5-10 cm) liver abscess/abscesses, liquified, drainable and divided randomly into two groups with 30 patients each and subjected to either Ultrasound-guided needle aspiration or catheter drainage with identical medical treatment. Outcomes were compared within both groups concerning the need for analgesics, total duration of hospital stay, total days of leave from work and recurrence or residual collection.

Results

Both groups were comparable in age, gender, type of abscess and maximal diameter. The success rate was equal (80% and 84%). However, the need for analgesics, total duration of hospital stay and total days of leave from work showed a significant (p<0.05) decrease in the needle aspiration group with a mean stay of 9.3 ± 3.18 days and mean leave of 18.9 ± 5.13 days as compared to catheter drainage group with the mean of 14.8 ±5.95 days and 32.5 ±11.4 days respectively.

Conclusion

Based on our study's results, we conclude that percutaneous needle aspiration is a primary interventional treatment in moderate-sized (5-10 cm) liver abscesses. More multicentric and randomised trials should be done to confirm the inference of this study.

## Introduction

Since ancient times, liver abscess has been troubling mankind as mentioned by Bhrigu-Samhita (dated 3000 B.C.) and Hippocrates (circa 400 B.C.). India has the second-highest incidence of liver abscesses in the world [1,2]. Historically liver abscess was treated by operative drainage as originally described by Volkmann in 1879 [3]. The mortality was as high as 75%-80% due to delayed presentation and treatment (mortality rate was as high as 80 to 90% if left untreated) while today, mortality is markedly decreased ranging from 10% to 20% due to improvements in various diagnostic and therapeutic procedures, invent of metronidazole, broad-spectrum antibiotics and radiology-guided drainage procedures [4,5].

Larger amoebic liver abscesses (ALA) with or without complications require intervention in the form of either closed or open drainage. However, there is no clear-cut consensus for the management of uncomplicated symptomatic medium-sized (5-10 cm) abscesses with treatment modalities ranging from drugs alone to needle aspiration to catheter drainage. The present study is an effort to compare the efficacy between percutaneous needle aspiration and pigtail drainage in medium-sized (5-10 cm) liver abscesses.

## Materials and methods

**Figure 1:**
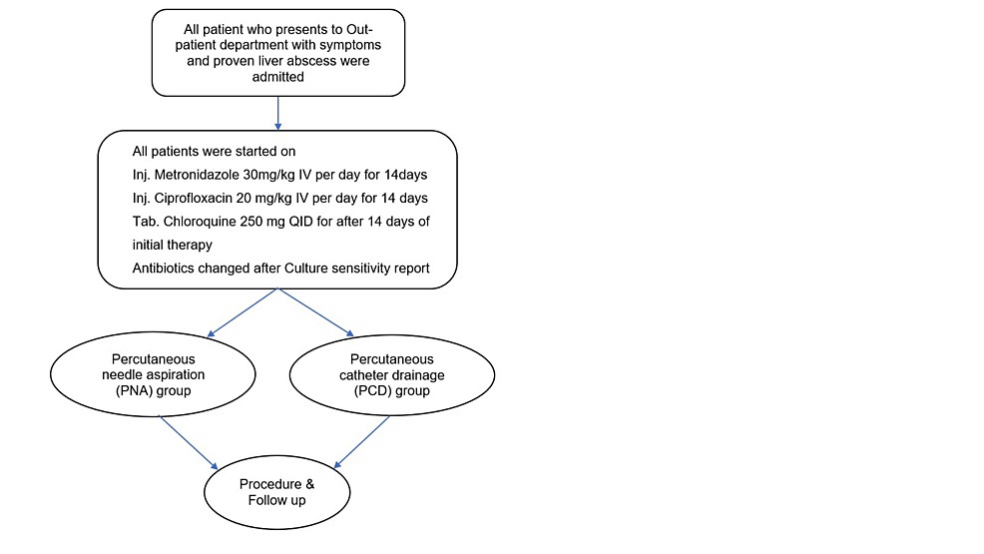
Schematic representation of study design IV - Intravenous QID - Quater in Die Inj - Injection Tab - Tablet

This study was a single tertiary‑centre prospective randomised comparative study and included a total of 60 patients with liquified liver abscesses on ultrasonography (USG) after approval of the study design by the institutional ethics committee. These patients were randomised into two groups: percutaneous needle aspiration (PNA) (n = 30) and percutaneous catheter drainage (PCD) (n = 30) after screening and written informed consent. Randomisation was done with a computer-generated randomisation chart. All the patients received the same medical therapy as shown in Figure 1. Chloroquine was started in all patients after checking G6PD (Glucose-6-phosphate dehydrogenase) levels because of the high prevalence of amoebiasis in our population to prevent recurrences. Exclusion criteria were all abscess cavities smaller than 5 cm and larger than 10 cm in their greatest dimension, pregnancy, prior intervention, ruptured liver abscess, and age less than 18 and more than 60 years. A total of 69 patients were screened, after assessment five patients were excluded due to larger-sized (>10cm) abscesses and four due to rupture.

Percutaneous needle aspiration

The subjects were evaluated with USG of the abdomen and the characteristics of the abscess cavities were recorded. Local anaesthesia was infiltrated at the puncture site with a 23 G needle. Under real-time USG guidance, using a 16 G BD spinal needle the abscess cavity was entered and pus was aspirated till no more pus could be aspirated further. A sample of pus was sent for microbial cultures. Gram stain and wet mount for *Entamoeba histolytica* trophozoites. A sterile dressing was applied at the needle puncture site. Every fifth-day review USG is performed and aspiration is repeated if there is less than a 50% reduction in the size of the abscess cavity or the presence of aspirable pus.

Percutaneous catheter drainage

The patient was subje­cted to USG of the abdomen and the characteristics of the abscess cavities were recorded. Local anaesthesia was infiltrated at the puncture site with a 23 G needle. Under real-time USG guidance, using the trocar method with a 14-French multiple-side hole pigtail catheter was introduced into the abscess cavity. Careful localisation of the abscess and proper selection of the entry site was required. Aspiration was done until no more pus was removed. The catheter was then secured to the skin for continuous external drainage and left in place until the cavity was drained. On every fifth day, residual cavities of abscesses were assessed by ultrasound examination and managed by catheter repositioning and aspiration or by the introduction of a new catheter into another cavity.

All patients underwent clinical follow-up, monitoring for clinical improvement and the requirement of analgesics during daily rounds until they were discharged from the hospital. Follow-up USG was performed every fifth day, and the size of the abscess was recorded. Criteria for successful treatment were clinical subsidence of infection, symptoms and sonographic evidence of abscess resolution, such as disappearance or marked decrease in the abscess cavity (more than 50% reduction of longest diameter before treatment).

After discharge from the hospital, patients underwent follow-up evaluations in the outpatient clinic every two weeks till six weeks after discharge. The patient outcome, including length of hospital stay, complications related to the procedure and treatment failure were recorded.

## Results

Both the groups were comparable in basic characteristics as shown in Table 1. The mean age was 39.2 ± 9.8 years, male to female ratio was 3.64:1 suggestive of the preponderance of disease in males due to exposure to multiple factors with a p-value of 0.012 (<0.05). Out of 60 patients, 44 (73%) had ALA and 16 (27%) had pyogenic liver abscesses based on the pus characteristics and the culture report, the ratio was 2.7:1 (A:P).

**Table 1:** Basic characteristics of the two arms PCD - percutaneous catheter drainage PNA - percutaneous needle aspiration

	Age in years				Men	Women	Alcoholics	Diabetics	Abscess type	
	18-30	31-40	41-50	51-60					Pyogenic	Amoebic
PCD Group	5	9	12	4	24	6	20	7	10	20
PNA Group	7	11	9	3	23	7	18	5	6	24
P value	0.77				0.75		0.59	0.51	0.24	

There was a positive association of alcoholism with ALA with an odds ratio of 2.4. In our study, acute appendicitis was associated with two patients with pyogenic liver abscesses.

Seventy-one per cent of patients had right lobe abscesses and the commonest presentation was abdominal pain (96%) followed by fever (66%).

Haemoglobin distribution shows ALA was prevalent in non-anaemic patients as iron deficiency anaemia confers some degree of protection against ALA.

**Figure 2:**
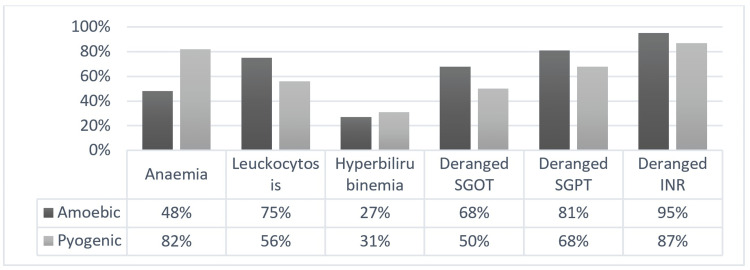
Laboratory data distribution based on the type of abscess SGOT - serum glutamic oxaloacetic transaminase SGPT - serum glutamate pyruvate transaminase INR - International Normalised Ratio

The most common parameter deranged in both abscesses is International Normalised Ratio (INR) followed by raise in serum glutamate pyruvate transaminase (SGPT) (Figure 2).

The mean diameter in the PCD group was 7.78 ± 0.96cm and the PNA group was 7.41 ± 1.17 cm with a p-value of 0.18. It was found that all the patients from the catheter drainage group required analgesia for more than three days post-procedure and on the contrary, no patient required analgesia for more than three days in the PNA group.

**Figure 3:**
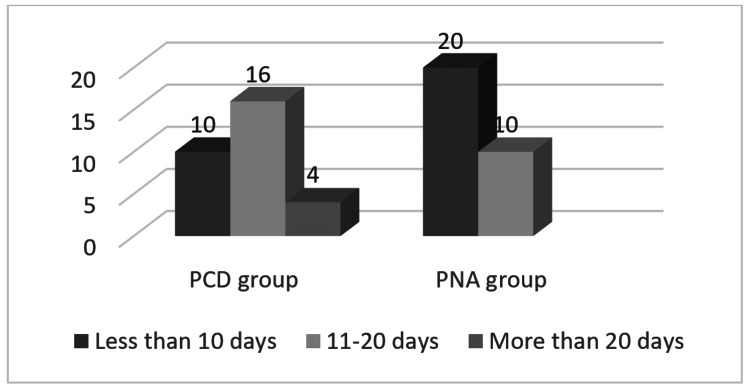
Comparison of total days of hospital stay in both study groups PCD - percutaneous catheter drainage PNA - percutaneous needle aspiration Y axis - number of patients

The mean total duration of hospital stay was 14.8 ± 5.95 days in the PCD group and 9.3 ± 3.18 days in the PNA group (Figure 3). Showed a statistically significant reduction in the PNA group with a p-value of 0.013.

**Figure 4:**
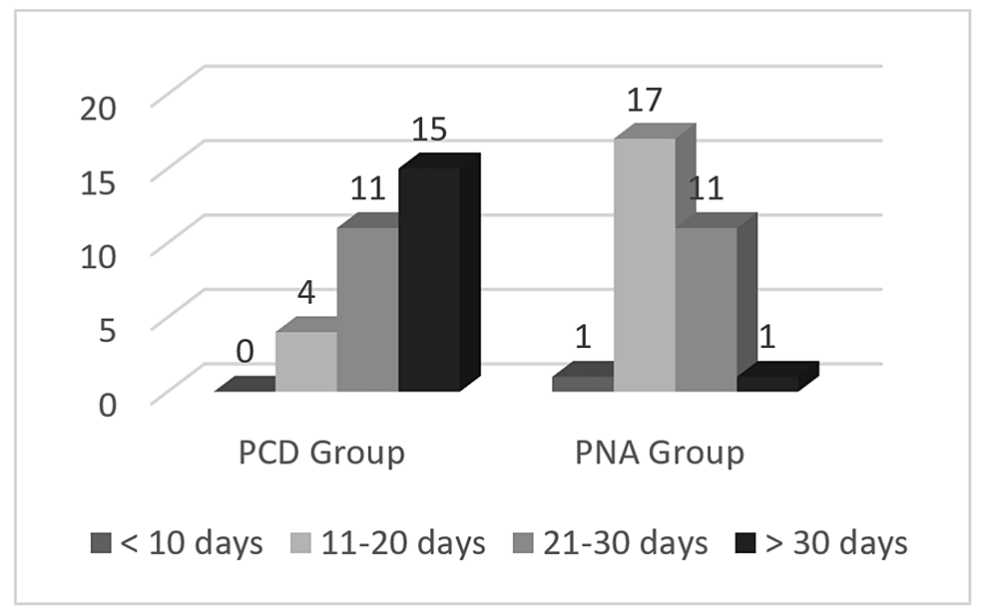
Comparison of total days of leave from work in both study groups PCD - percutaneous catheter drainage PNA - percutaneous needle aspiration Y axis - number of patients

The mean total duration of leave from work observed was 32.5 ± 11.4 days in the PCD group and 18.9 ± 5.13 days in the PNA group (Figure 4). The chi-square test showed a statistically significant difference between the two groups with a p-value <0.001.

Despite the above differences, the outcome on the sixth-week follow-up for recurrence or residual liver abscess was found to be five and six recurrences in PCD and PNA groups respectively, without any statistical significance p-value = 0.73.

## Discussion

In developed countries, three-quarters of liver abscesses are pyogenic abscesses whereas in developing countries the majority are ALA. The incidence is high in some tropical countries and it is attributed to a lack of proper sanitation and personal hygiene due to low socio-economic conditions. Amoebiasis affects approximately 50 million cases and 100,000 deaths annually all over the world. India has the second-highest incidence of liver abscesses in the world [1,6]. ALA is the most common (3-9%) extra intestinal manifestation of amoebiasis [2]. The majority of our study subjects were from the low socio-economic group. In this era of minimally invasive treatment, liver abscesses treated with a combination of IV antibiotics and percutaneous procedures (PCD and PCN) show variable degrees of success and reduced mortality and morbidity compared to open drainage. Whether to prefer needle aspiration or catheter drainage continues to be debated especially in underdeveloped and developing countries where not all patients can afford to undergo costly procedures. This study aimed to establish a preferable method for the treatment of medium-sized (5-10 cm) abscesses.

In a study, Debakey and Ochsner et al., in 1951, studied 263 cases and found that the incidence was maximum in the 31-40 years of age group. The present study shows the common age group of liver abscess as 30-50 years recent studies conducted in India also contemplated the same [3,7-9].

Male to female ratio in the present study was 3.6:1, like recent studies conducted in India by Onkar Singh et al., Tekam Vijay Kumar et al. and Mukesh Kulhari et al. [5,8,10]. In the present study, alcohol was the single most consistent etiological factor of liver abscess especially amoebic compared to Ajay Chauhan et al., Choudhary et al. and Kumar et al. [2,11]. Lobar involvement of liver abscess in our study was 71.7% in the right lobe, 18.3% in the left lobe and bilobar involvement was seen in 10% of the subjects comparable with recent studies by Farman Ali et al., Paramdeep Singh et al., Tekam Vijay Kumar et al. and Kumar et al. [1,5-7].

Like recent studies, the commonest presenting complaint in the present study was abdominal pain followed by fever, in the laboratory profile of the study subjects high haemoglobin concentration was associated with a high incidence of ALA as shown in previous studies as a protective factor for women with iron deficiency anaemia [8,10,12].

Intravenous analgesic requirement for more than three days was observed in all the patients in the PCD group as compared to none in the PNA group who required IV analgesics which can be attributed to the presence of the catheter in situ. The mean total duration of hospital stay noted in our study was 14.8 ± 5.95 days in the PCD group and 9.3 ± 3.18 days in the PNA group consistent with recent studies with a p-value of 0.013 [2,9] and the mean total duration of leave from work observed in two groups were 32.5 ± 11.4 days in PCD group and 18.9 ± 5.13 days in PNA group which shows a statistically significant reduction in PNA group with a p-value of <0.001.

The pain related to in situ catheter causes delayed mobilisation and leads to an increased hospital stay with the delayed resumption of normal activities. The main reason was the stretch of the catheter and psychosocial issues of the patient due to the presence of the catheter and drainage bag as observed in our study by interviews with patients. The majority of our patients are from lower and middle socio-economic classes and absence from work made a high impact on their quality of life.

Our study found PNA is equally efficacious to catheter drainage in treating moderate-size liver abscesses (5-10 cm in diameter). It was better, effective, easier, simpler, less time-consuming and less expensive. The reduced analgesic requirement and early discharge from the hospital in the needle aspiration group were beneficial to patients as they could resume their work as compared to the catheter drainage group.

Limitations of this study were the small number of patients studied (60 patients) and the shorter duration of follow-up. Multicentric and randomised trials should be done to confirm the inference of this study.

## Conclusions

Liver abscesses are common pathology encountered worldwide and drainage of the abscess cavity forms a major component of the treatment. Based on the results of our study we conclude PNA is a primary interventional treatment in moderate-sized (5-10 cm) liver abscesses in terms of advantages like reduced pain, early discharge and early return to work.
